# Inhaled Treprostinil: Improvements in Hemodynamics and Quality of Life for Patients with Pulmonary Arterial Hypertension on Dual or Triple Therapy

**DOI:** 10.3390/jcm14248776

**Published:** 2025-12-11

**Authors:** Shogo Ikegami, Takahiro Hiraide, Takashi Maeda, Mizuki Momoi, Yoshiki Shinya, Atsushi Anzai, Yasuyuki Shiraishi, Yoshinori Katsumata, Masaki Ieda

**Affiliations:** 1 Department of Cardiology, Keio University School of Medicine, Tokyo 160-8582, Japan; 2 Institute for Integrated Sports Medicine, Keio University School of Medicine, Tokyo 160-8582, Japan

**Keywords:** KCCQ, treatment response, inhaled treprostinil

## Abstract

**Background:** Pulmonary arterial hypertension (PAH) leads to right ventricular failure and death. Inhaled treprostinil, a tricyclic benzindene prostacyclin analog, has become available, but evidence regarding its clinical efficacy and quality-of-life (QoL) benefits—particularly in patients already receiving optimized combination vasodilator therapy—remains limited. **Methods:** Inhaled treprostinil was introduced to nine patients with PAH already receiving combination therapy with pulmonary vasodilators. Acute hemodynamic effects were assessed during initial right heart catheterization, and long-term effects were evaluated at baseline and 3 months after treatment. Exercise tolerance was assessed by the 6-minute walking distance (6MWD) test and cardiopulmonary exercise testing, while QoL was evaluated using the Kansas City Cardiomyopathy Questionnaire-12 (KCCQ-12). **Results:** Mean pulmonary arterial pressure significantly improved both acutely (48.9 ± 17.8 to 43.7 ± 14.5 mmHg, *p* = 0.036) and at 3 months (46.4 ± 16.1 to 39.8 ± 14.1 mmHg, *p* = 0.014). Pulmonary vascular resistance tended to decrease, while 6MWD outcomes remained unchanged. QoL improved, with KCCQ-12 overall and clinical summary scores increasing from 59.1 ± 27.4 to 67.1 ± 26.5 and 78.1 ± 26.3 to 87.5 ± 21.2, respectively. **Conclusions:** Treprostinil inhalation improved hemodynamics and patient-reported outcomes despite prior combination improved hemodynamics and tended to enhance QoL in patients with PAH receiving combination vasodilator therapy.

## 1. Introduction

Pulmonary arterial hypertension (PAH) is a rare and progressive disease characterized by elevated vascular resistance, which leads to right ventricular failure and death [1]. This disease typically manifests in early adulthood and historically carried a median survival of only 2.8 years in the absence of treatment [2]. However, life expectancy has improved markedly with the development of several pulmonary vasodilators, such as prostaglandin I_2_ (PGI_2_) analogs, PGI_2_ receptor agonists, phosphodiesterase 5 (PDE5) inhibitors, soluble guanylate cyclase (sGC) stimulators, and endothelin receptor antagonists (ERAs) [1]. Contemporary management strategies guided by evidence-based risk stratification have further contributed to improved outcomes.

Inhaled treprostinil, a tricyclic benzindene prostacyclin analog, became available in Japan in 2023. When added to background therapy with ERAs and PDE5 inhibitors or sGC stimulators, inhaled treprostinil has been shown to improve hemodynamics and exercise capacity [3,4]. Consequently, current guidelines recommend its use in patients who remain at intermediate–low risk after initial dual combination therapy [1,5]. However, evidence remains limited for the use of inhaled treprostinil in intermediate–high- and high-risk patients following combination therapy. Moreover, the therapeutic landscape of PAH continues to evolve, with increasing use of potent agents such as selexipag and the growing adoption of upfront triple therapy. As treatment strategies become more intensive, clinicians are increasingly confronted with a practical clinical dilemma: does inhaled treprostinil retain a meaningful therapeutic role in patients who are already receiving optimized combination therapy yet remain symptomatic?

Quality of life (QoL) has emerged as an important treatment goal in modern PAH management, reflecting the prolonged life expectancy achieved with current therapies [1,6]. Previous studies have demonstrated that inhaled PGI_2_ analogs, such as iloprost, improve hemodynamics and QoL [7,8], and inhaled treprostinil similarly improves 6 min walking distance (6MWD), and QoL in patients with PAH [9,10,11] and those with pulmonary hypertension associated with interstitial lung disease [12]. Nevertheless, evidence remains limited regarding the real-world effectiveness of inhaled treprostinil—particularly its impact on QoL—in patients who are already well treated with combination vasodilator therapy. This gap is clinically relevant, as many patients continue to experience symptoms or impaired QoL despite achieving hemodynamic improvement or stabilization with current combination therapy of pulmonary vasodilators.

In this study, we evaluated the effect of inhaled treprostinil on hemodynamics and QoL in patients receiving combination therapy for PAH in a real-world clinical setting.

## 2. Materials and Methods

### 2.1. Patients and Measurements

This was a single-center, prospective observational study conducted at Keio University Hospital. We included all patients who initiated inhaled treprostinil between July 2023 and February 2024. We enrolled nine patients with PAH in WHO Group 1 who had already been receiving combination therapy for at least one year. Risk stratification was performed in accordance with the current guideline [1]. Among the participating patients, four were classified as low or low–intermediate risk with relatively stable hemodynamics but persistent exertional dyspnea despite combination therapy. The remaining five patients were clinically stable but classified as intermediate–high or high risk. Inhaled treprostinil was selected because parenteral prostacyclin therapy is considered challenging. No patients were excluded during the screening due to intolerance, oxygenation issues, or other safety concerns. The dosage of treprostinil was titrated according to the manufacturer’s protocol. We performed right heart catheterization (RHC) at the initiation of treprostinil, and all patients underwent follow-up RHC approximately 3 months later during the trough phase. We also assessed the patients’ backgrounds, and histories of treatment with 6MWD, transthoracic echocardiography, and B-type natriuretic peptide concentrations. Moreover, we conducted cardiopulmonary exercise testing on six patients and evaluated their peak oxygen uptake before and after the use of inhaled treprostinil.

This study was approved by the Ethics Committee of Keio University Hospital (#20140203), and all patients provided written informed consent. This study was conducted in accordance with the principles of the Declaration of Helsinki.

### 2.2. RHC

RHC was performed from the right jugular vein using a 7-Fr Swan-Ganz catheter (Edwards Lifesciences, Irvine, CA, USA). All procedures were carried out by experienced operators following standardized institutional protocols to ensure the consistency of the measurements. Cardiac output (CO) was calculated using the direct Fick equation; in patients with home oxygen therapy, the thermodilution method was used. Pulmonary vascular resistance (PVR) was calculated as follows: mean pulmonary arterial pressure (PAP) minus mean pulmonary artery wedge pressure (PAWP) divided by CO. Hemodynamic parameters at baseline were analyzed at 5, 10, 15, and 20 min after three inhalations of treprostinil. We performed RHC approximately 3 months after the initiation of treprostinil inhalation. RHC was performed during the trough phase of inhaled treprostinil therapy (4 h after the last inhalation).

### 2.3. QoL Analysis

To assess QoL, we evaluated the Kansas City Cardiomyopathy Questionnaire-12 (KCCQ-12) scores at baseline and 3 months later. The KCCQ-12 is a validated questionnaire for left-sided heart failure. The KCCQ-12 was chosen for this study because of its established reliability in cardiovascular diseases, such as heart failure with preserved ejection fraction [13,14] and right heart failure [15]. We acknowledge that its sensitivity to change in PAH may be somewhat limited; however, it captures clinically relevant domains of physical limitation, symptom burden, and overall wellbeing, making it a practical instrument for patient-reported outcomes in this setting. This questionnaire was composed of five parts: clinical summary score (KCCQ-CSS), physical limitation (KCCQ-PL), symptom frequency (KCCQ-SF), quality of life (KCCQ-QL), and symptom limitation (KCCQ-SL). KCCQ-CSS is calculated as the average of the KCCQ-PL and KCCQ-SF score. All KCCQ-12 scores scale from 0 to 100 are frequently summarized using the following 25-point ranges: 0 to 24—very poor–poor; 25 to 49—poor–fair; 50 to 74—fair–good; 75 to 100—good–excellent.

### 2.4. Statical Analysis

Continuous variables are presented as the mean ± standard deviation or as the median (25th–75th percentile range), while categorical variables are presented as counts and percentages. We evaluated hemodynamic changes during the first RHC after the inhalation of treprostinil, and compared the changes between the baseline and at 20 min. Changes in hemodynamics, QoL and other variables from baseline to 3 months were also evaluated. Additionally, we compared the changes in hemodynamics and KCCQ-12 scores between low/intermediate–low-risk patients and intermediate–high/high-risk patients. All comparisons were performed using the Wilcoxon signed-rank test, and *p* values < 0.05 were considered statistically significant. In the KCCQ-12 analysis, a change of 5 points was considered clinically meaningful, while a change of 10 points represented a moderate-to-large clinical change [13]. All analyses were conducted using R software version 4.4.0 (R Foundation for Statistical Computing, Vienna, Austria).

## 3. Results

### 3.1. Baseline Characteristics

The baseline characteristics of the nine patients are shown in Table 1. The mean age was 53.1 ± 15.1 years, and two-thirds were women. Eight patients were receiving combined therapy including a PGI_2_ receptor agonist (selexipag) at baseline. According to guideline-based risk stratification, four patients were identified as having a low/intermediate–low risk of PAH after the initial combination therapy of pulmonary vasodilators, and five were identified as having an intermediate–high/high risk. In two high-risk patients (#7 and #9) parenteral prostacyclin therapy was not feasible, and inhaled treprostinil was selected as an alternative. No patients were excluded during the screening.

We maintained treatment adherence through monthly outpatient visits, during which physicians and nurses reviewed and reinforced the patient’s inhalation technique. At 1 month after the initiation of inhaled treprostinil, pharyngeal discomfort was reported in 7 patients (78%), cough in 6 patients (67%), and headache in 3 patients (33%). By 6 months, these symptoms had substantially decreased, with pharyngeal discomfort observed in 2 patients (22%), cough in 3 patients (33%), and headache in 2 patients (22%). Other adverse events, including nasal congestion, throat irritation, and gastrointestinal symptoms, were infrequent and generally resolved over time.

### 3.2. Acute Response of Inhaled Treprostinil

**Figure 1:**
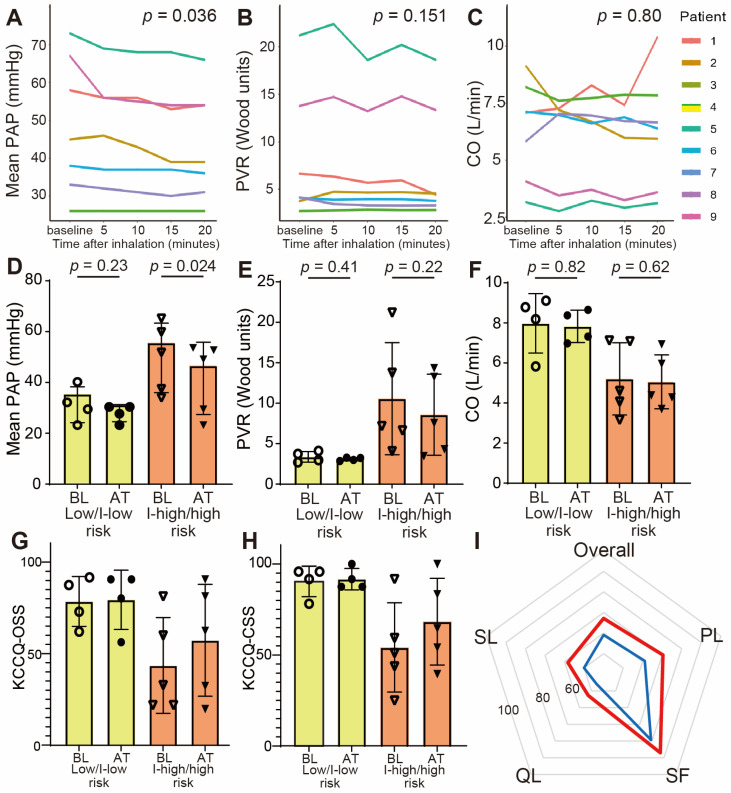
Changes in hemodynamic parameters and QoL after treprostinil inhalation treatment. Individual changes in (**A**) mean pulmonary arterial pressure (PAP), (**B**) pulmonary vascular resistance (PVR), (**C**) cardiac output (CO) up to 20 min after inhalation. (**D**–**F**) Changes in mean PAP, PVR, and CO were compared between low/intermediate–low (I-low)-risk patients (yellow boxes) and intermediate–high (I-high)/high-risk patients (orange boxes) at baseline (BT) and after the treatment (AT). BNP concentrations are shown in a logarithmic scale. Patients 7 and 9 could not have the 6MWD measured at baseline because of dyspnea at rest. (**G**,**H**) Changes in KCCQ overall summary score (KCCQ-OSS) and clinical summary score (KCCQ-CSS) were compared according to the risk stratification. (**I**) A radar chart illustrating the changes in KCCQ subcategories before and after the treatment. Blue represents baseline, and red represents post-treatment. PL, physical limitation; QL, quality of life; SF, symptom frequency; SL, symptom limitation.

We evaluated the acute response of inhaled treprostinil in seven patients (Figure 1A–C and Supplementary Materials Figure S1A–C). Mean PAP significantly decreased from 48.9 ± 17.8 mmHg to 43.7 ± 14.5 mmHg (*p* = 0.036), and PVR improved from 8.0 ± 6.9 Wood units to 7.3 ± 6.2 Wood units (*p* = 0.151) 20 min after the inhalation. CO, PAWP, systemic blood pressure, and oxygen saturation remained stable, indicating that acute vasodilation occurred without compromising systemic hemodynamics. Although the extent of improvement varied among individuals, the overall pattern suggested a consistent acute vasodilatory effect.

### 3.3. Hemodynamic Changes at 3 Months

Inhaled treprostinil was titrated to the maximum dose except in patient 1. Patient 1 had 12 inhalations each day and could not have the dose titrated because of a headache and fatigue. The changes in hemodynamics at baseline and after 3 months are shown in Figure 1D–F, Supplementary Materials Figure S1D–I, and Table 2. Mean PAP decreased from 46.4 ± 16.1 mmHg to 39.8 ± 14.1 mmHg (*p* = 0.014), and PVR also demonstrated a numeric reduction from 7.4 ± 6.2 Wood units to 6.0 ± 4.7 Wood units (*p* = 0.124). Improvements were observed in both risk strata, although patients in the intermediate–high/high-risk group exhibited a more pronounced reduction in mPAP (*p* = 0.024). Cardiac output and PAWP showed no significant changes. BNP values tended to decrease, though not significantly. Echocardiographic measures, including LV ejection fraction and RV fractional area change, remained stable, consistent with the preserved biventricular function. Importantly, no patient experienced hemodynamic deterioration during the observation period.

### 3.4. Exercise Tolerance

Exercise tolerance remained stable during treatment. The mean 6 min walk distance was unchanged (464 ± 116 m at baseline and 464 ± 114 m at 3 months). In six patients without home oxygen therapy, cardiopulmonary exercise testing demonstrated mild improvements in peak oxygen uptake and ventilatory efficiency, although these changes were not statistically significant. These findings paralleled the modest but consistent hemodynamic improvements.

### 3.5. Quality of Life

The changes in KCCQ scores are shown in Figure 1G–I and Supplementary Materials Figure S2A–J. Although the combination therapy of pulmonary vasodilators had already started, the KCCQ-12 overall summary score increased from 59.1 ± 27.4 to 67.1 ± 26.5 (*p* = 0.097) and KCCQ-CSS improved from 78.1 ± 26.3 to 87.5 ± 21.2, 3 months after the initiation of inhaled treprostinil (*p* = 0.109). In the other domains, there were improvements of more than 5 points in the KCCQ-PL (61.1 ± 31.5 to 70.4 ± 28.9, *p* = 0.191), KCCQ-SF (79.4 ± 22.2 to 87.0 ± 16.6, *p* = 0.281), and KCCQ-SL (50.0 ± 35.4 to 58.3 ± 37.7, *p* = 0.800); these were clinically significant improvements. The KCCQ-QL was slightly improved from 50.0 ± 30.0 to 52.8 ± 32.3 (*p* = 0.590). In patients with an intermediate–high/high risk, all domains were improved by more than 10 points, which suggested clinically important change of QoL after treprostinil inhalation treatment.

## 4. Discussion

This study showed that inhaled treprostinil significantly improved hemodynamics in the acute phase and during the trough phase after 3 months, especially in intermediate–high/high-risk patients, in addition to the combination therapy of pulmonary vasodilators including prostacyclin receptor agonists. Almost all patients tolerated the maximum dose of inhaled treprostinil without any obvious adverse effects, and a significant improvement in QoL was observed especially in patients with an intermediate–high/high risk. Importantly, our findings suggest that inhaled treprostinil may help restore a therapeutic window in patients with more advanced disease who remain symptomatic despite intensive oral therapy and who are not receiving parenteral prostacyclin analogues.

At the Seventh World Symposium on Pulmonary Hypertension held in 2024, the use of inhaled treprostinil was recommended for patients classified as intermediate–low-risk on the basis of a four-strata risk assessment following initial combination therapy with an ERA and PDE5 inhibitor [5]. Inhaled treprostinil improves the 6MWD in patients with pulmonary arterial hypertension [3]. Additionally, a previous Japanese study showed a hemodynamic improvement in patients who were predominantly classified as intermediate–low-risk after receiving inhaled treprostinil [7]. The use of intravenous or subcutaneous prostacyclin analogs is the gold standard for patients classified as intermediate–high or high risk, but these therapies are not feasible for some patients owing to frailty, catheter-related infection risk, or patient preference. This suggests that inhaled therapy may be considered as an alternative option in individuals for whom parenteral prostacyclin therapy is challenging, such as older patients or those at high risk of catheter-related infections. In most cases included in this study, the patients were already receiving a triple oral therapy regimen consisting of an ERA, PDE5 inhibitor or sGC stimulator, and selexipag. Treprostinil is a prostacyclin analog, whereas selexipag is a prostacyclin receptor agonist, with slightly different mechanisms of action. Previous studies on inhaled treprostinil have primarily focused on its addition to ERAs and PDE5 inhibitors or sGC stimulators [4,7]. Recent real-world analyses have also supported the clinical utility of inhaled treprostinil. Balakrishnan et al. reported that treatment response and hemodynamic improvement vary according to baseline characteristics, highlighting the importance of patient selection in maximizing benefit [16]. In addition, the initiation of inhaled treprostinil in clinical practice was associated with reductions in hospitalization and intensive care utilization among patients with pulmonary hypertension with lung disease [17]. These findings further reinforce the potential role of inhaled treprostinil across diverse patient populations. Furthermore, a recent real-world study from the SPHERE registry demonstrated that even patients receiving optimized selexipag therapy often remain symptomatic and require additional treatment escalation [18]. Our study extends these findings by demonstrating that inhaled treprostinil provided additional hemodynamic and QoL benefits even in patients already treated with highly titrated selexipag, consistent with studies that reported an improvement in hemodynamics when selexipag was added to parental prostacyclin therapy [19]. Our results suggest that, in patients who do not achieve a low-risk profile despite the addition of selexipag, further augmentation of the prostacyclin pathway with inhaled treprostinil may represent a viable therapeutic option.

Regarding QoL, KCCQ-12 scores were improved in all domains, especially in patients with an intermediate–high or high risk. There are multiple QoL measures, such as the Emphasis-10 [20], the Medical Outcomes Study 36-Item Short-Form Health System [21], and Pulmonary Arterial Hypertension-Symptoms and Impact [22]. We analyzed the KCCQ-12 score because symptoms in patients with PAH are related to chronic right heart failure, and the KCCQ is a key QoL measurement in patients with PAH [22]. Physical limitations and the frequency of symptoms were greatly improved 3 months after the initiation of inhaled treprostinil, even in intermediate–high-/high-risk patients. Given that acute hemodynamic improvement was observed within 20 min of inhalation, it is plausible that early enhancement in exercise capacity may have contributed to the reduction in physical limitations and symptom burden.

At follow-up, the mean PAP was significantly reduced even during the trough phase of inhaled treprostinil. A previous study demonstrated that improvements in mean PAP were closely associated with reductions in mortality risk through improvements in risk-stratification scores [23]; therefore, the sustained decrease in mean PAP observed in our cohort may reflect not only symptomatic improvement but also a shift toward a more favorable risk profile.

KCCQ-QL scores were also improved, especially in patients with an intermediate–high-/high risk. Continuous administration of parental PGI_2_ analogs requires careful management, including infection control and pain control, and this may limit the patients’ QoL. Therefore, the introduction of inhaled treprostinil could be a treatment option in patients with an intermediate–high-/high risk who face challenges in managing parental prostacyclin analogs to improve hemodynamics and QoL. Furthermore, patient preference and the ability to adhere to inhaled therapy are important considerations, as real-world success often depends more on treatment acceptability and inhalation burden than on physiological effects alone.

This study has some limitations. First, this was a single-center, retrospective study with a small sample size, thus stratified analyses according to age or disease severity were difficult to perform. Second, we performed a single-arm trial, so the placebo effects of inhalation cannot be ruled out; however, peak oxygen uptake in cardiopulmonary exercise testing tended to improve at follow-up. Therefore, we believe that the improvement in KCCQ scores is attributable to the medication. Third, we did not use multiple QoL indicators. KCCQ, which is the most reliable indicator of heart failure, was used in our study; however, its sensitivity and applicability in PAH may be limited. Fourth, we measured the patients’ hemodynamics at the trough phase of inhaled treprostinil therapy, but did not assess the acute changes in hemodynamics at follow-up. However, this study showed that mean PAP was significantly improved, even at the trough phase, after treatment, suggesting the effectiveness of this treatment. Fifth, the relatively short follow-up restricts the assessment of long-term effects. Finally, we acknowledge that the absence of a control group and the observational study design limit any causal interpretation of our findings.

## 5. Conclusions

Inhaled treprostinil appears to be a promising addition to combination therapy for PAH, particularly for patients at intermediate–high risk. Larger, multicenter studies are required to confirm these results and further evaluate the long-term benefits of inhaled treprostinil.

## Figures and Tables

**Table 1:** Baseline characteristics of the patients.

Patient	Age (Years)	Sex	Diagnosis	BMI (kg/m^2^)	WHO-FC	ESC/ERS Risk Stratification	Medications	BNP (pg/mL)	RV FAC (%)	Mean PAP (mmHg)	PVR (Wood Units)	CO (L/min)	PAWP (mmHg)	SaO_2_ (%)	SvO_2_ (%)	6MWD (m)	KCCQ-12 Total Score
1	48	F	IPAH	25.8	III	intermediate–high	Macitentan 10 mg Tadatafil 40 mg Selexipag 3.2 mg	39.3	18	58	6.6	7.1	11	92.4 (**)	77.4 (**)	320	21.9
2	35	M	HPAH	25.1	II	low	Macitentan 10 mg Riociguat 7.5 mg Selexipag 3.2 mg	16.4	39.3	45	3.7	9.1	11	95.4	80.7	552	62
3	46	F	CHD-PAH *	20.4	II	intermediate–low	Macitentan 10 mg Tadatafil 40 mg Selexipag 3.2 mg	17.6	36.6	26	2.7	8.2	4	94.9	76.9	430	72.9
4	48	F	HPAH	21.2	III	intermediate–high	Macitentan 10 mg Tadatafil 40 mg	361.8	24.6	73	21.2	3.2	8	96.1	58.9	425	81.3
5	72	F	HPAH	21.3	III	intermediate–high	Macitentan 10 mg Riociguat 7.5 mg Selexipag 3.2 mg	297	36.4	38	4.1	7.1	9	93.1	77	388	59.9
6	44	M	IPAH	28.3	II	low	Macitentan 10 mg Riociguat 7.5 mg Selexipag 3.2 mg	6.8	28.9	33	4.1	5.8	8	97.4	74.2	455	87.5
7	69	F	IPAH	17.7	IV	high	Macitentan 10 mg Riociguat 7.5 mg Selexipag 3.2 mg	163.9	34.4	42	7.2	4.6	7	97.3	71.1	NA	32.8
8	40	F	IPAH	19.1	II	low	Macitentan 10 mg Riociguat 7.5 mg Selexipag 3.2 mg	23.7	36.3	36	3	8.8	10	95.3	78.7	675	91.7
9	76	M	HPAH	16.2	III	high	Macitentan 10 mg Riociguat 7.5 mg Selexipag 3.2 mg	294.5	18.2	67	13.8	4.1	8	97.6	73.8	NA	21.9

Abbreviations: BMI, body mass index; BNP, B-type natriuretic peptide; CO, cardiac output; CHD-PH, congenital heart disease-associated pulmonary hypertension; F, female; HPAH, heritable pulmonary arterial hypertension; IPAH, idiopathic pulmonary arterial hypertension; KCCQ-12, Kansas City Cardiomyopathy Questionnaire-12; M, male; PAP, pulmonary arterial pressure; PAWP, pulmonary arterial wedge pressure; PVR, pulmonary vascular resistance; RV FAC, right ventricular fractional area change; SaO_2_, blood oxygen saturation; SvO_2_, mixed venous oxygen saturation; WHO-FC, World Health Organization functional class; 6MWD, 6 min walking distance. * PAH after atrial septal defect correction. ** These data were obtained under supplemental oxygen at 2 L/min.

**Table 2:** Changes in hemodynamics, echocardiographic parameters, spirometry, and exercise tolerance.

	Baseline	After 3 Months	*p* Value
Mean BP (mmHg)	74.3 ± 7.4	81.2 ± 9.8	0.0972
Mean PAP (mmHg)	46.4 ± 16.1	39.8 ± 14.1	0.014
PVR (WU)	7.4 ± 6.2	6.1 ± 4.6	0.155
CO (L/min)	6.4 ± 2.1	6.3 ± 1.8	0.636
PAWP (mmHg)	8.4 ± 2.2	7.0 ± 2.1	0.105
SaO_2_ (%)	95.5 ± 1.9	93.5 ± 4.4	0.407
SvO_2_ (%)	74.3 ± 6.4	71.1 ± 7.3	0.075
BNP (pg/mL)	39.3 [17.6, 294.5]	22.3 [16.7, 163.1]	0.812
LVEF (%)	65.3 ± 10.0	68.1 ± 5.7	0.407
RVFAC (%)	30.3 ± 8.2	30.3 ± 9.1	1
6MWD (m)	464 ± 117	464 ± 114	1
peak VO_2_ (mL/kg/min)	16.0 ± 4.2	17.5 ± 5.6	0.419
VE/VCO_2_ slope	39.7 ± 5.5	38.1 ± 2.7	0.59
%VC	94.9 ± 9.2	95.7 ± 12.4	0.447
%FVC	94.8 ± 11.7	95.3 ± 12.1	0.8
FEV_1.0%_	67.2 ± 9.4	66.1 ± 5.6	0.554
%DLco	58.9 ± 11.7	60.8 ± 14.2	0.272
%DLco/VA	64.1 ± 12.2	65.5 ± 11.9	0.353

BNP concentrations are shown as the median [interquartile range] and other variables are shown as the mean ± standard deviation. BNP, B-type natriuretic peptide; BP, blood pressure; CO, cardiac output; HR, heart rate; LVEF, left ventricular ejection fraction; PAP, pulmonary arterial pressure; PAWP, pulmonary arterial wedge pressure; VO_2,_ oxygen uptake; PVR, pulmonary vascular resistance; RVFAC, right ventricular fractional area change; SaO_2_, blood oxygen saturation; SvO_2_, mixed venous oxygen saturation; 6MWD, 6 min walking distance; %DLco, percentage of diffusing capacity for carbon monoxide; %DLco/VA, percentage of diffusing capacity for carbon monoxide per unit of alveolar volume; %FVC, percentage of forced vital capacity; %VC, percentage of vital capacity.

## Data Availability

The data that support the findings of this study are available on request from the corresponding author.

## Supplementary Materials

The following supporting information can be downloaded at: https://www.mdpi.com/article/10.3390/jcm14248776/s1. Figure S1: Acute changes in hemodynamic parameters during baseline RHC. Figure S2: Changes in the Kansas City Cardiomyopathy Questionnaire-12 (KCCQ-12) score.

## Abbreviations

CO	cardiac output
ERA	endothelin receptor antagonists
KCCQ	Kansas City Cardiomyopathy Questionnaire
PAH	Pulmonary arterial hypertension
PAP	pulmonary artery pressure
PAWP	pulmonary artery wedge pressure
PDE5	phosphodiesterase 5
PVR	pulmonary vascular resistance
QoL	quality of life
RHC	right heart catheterization
sGC	soluble guanylate cyclase
6MWD	6 min walking distance
