# Supplementary material for: Inhaled Treprostinil: Improvements in Hemodynamics and Quality of Life for Patients with Pulmonary Arterial Hypertension on Dual or Triple Therapy
Source: J Clin Med. 2025 Dec 11;14(24):8776. doi: 10.3390/jcm14248776 (PMC12733909; doi:10.3390/jcm14248776)
Supplement: Supplementary file 1 [file jcm-14-08776-s001.zip › jcm-3973113-supplementary.pdf]

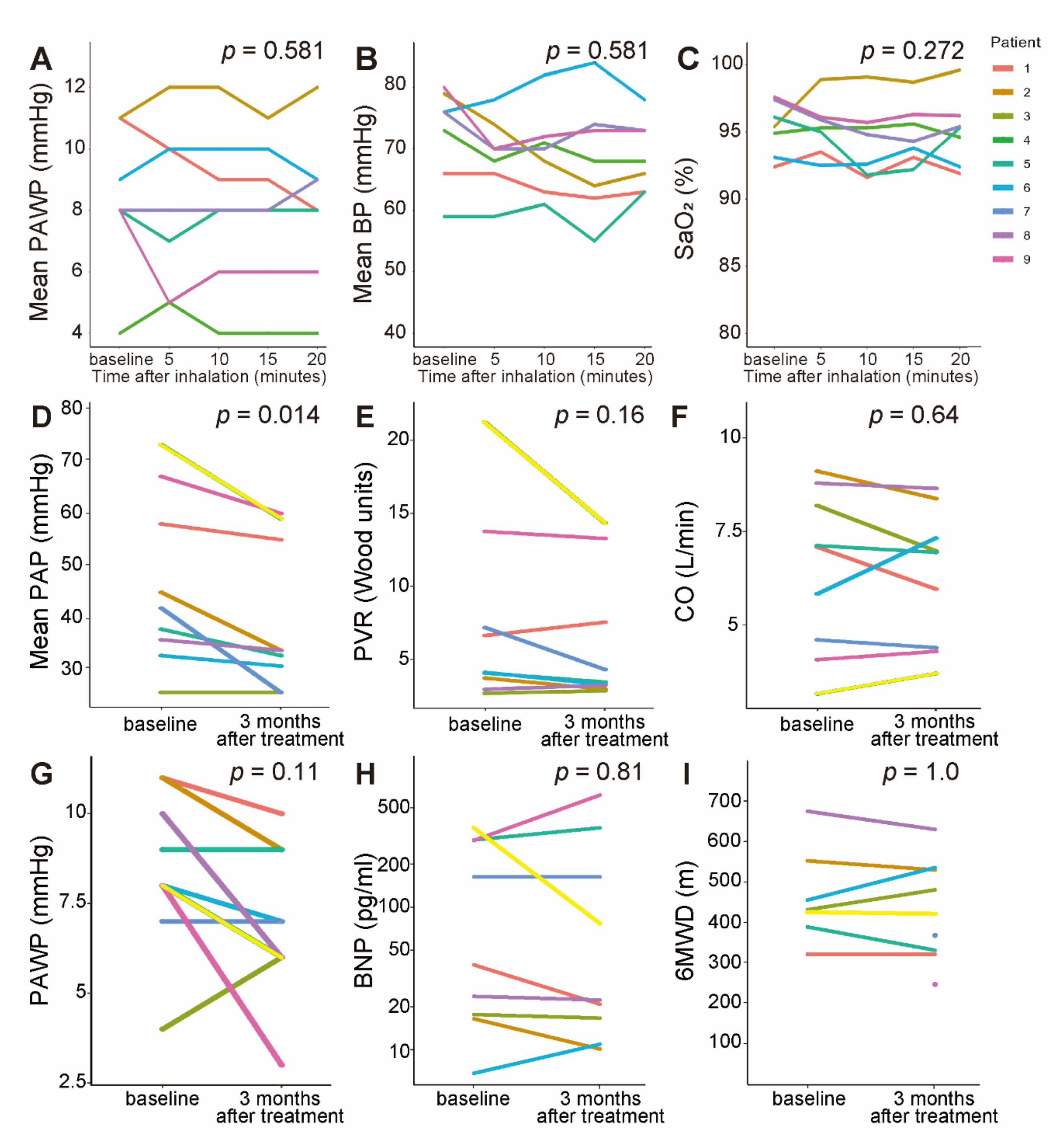


**Figure S1.** Acute changes in hemodynamic parameters during baseline RHC.

Individual changes in (A) pulmonary arterial wedge pressure (PAWP), (B) mean blood pressure (BP), and (C) blood oxygen saturation (SaO2) up to 20 minutes after inhalation. Individual changes in (D) mean pulmonary arterial pressure (PAP), (E) pulmonary vascular resistance (PVR), (F) cardiac output (CO), (G) PAWP, (H) BNP concentrations, and (I) 6MWD at baseline and follow-up for 3 months. BNP concentrations are shown in a logarithmic scale. Patients 7 and 9 could not have the 6MWD measured at baseline because of dyspnea at rest. Data of each patient are shown in separate colors.

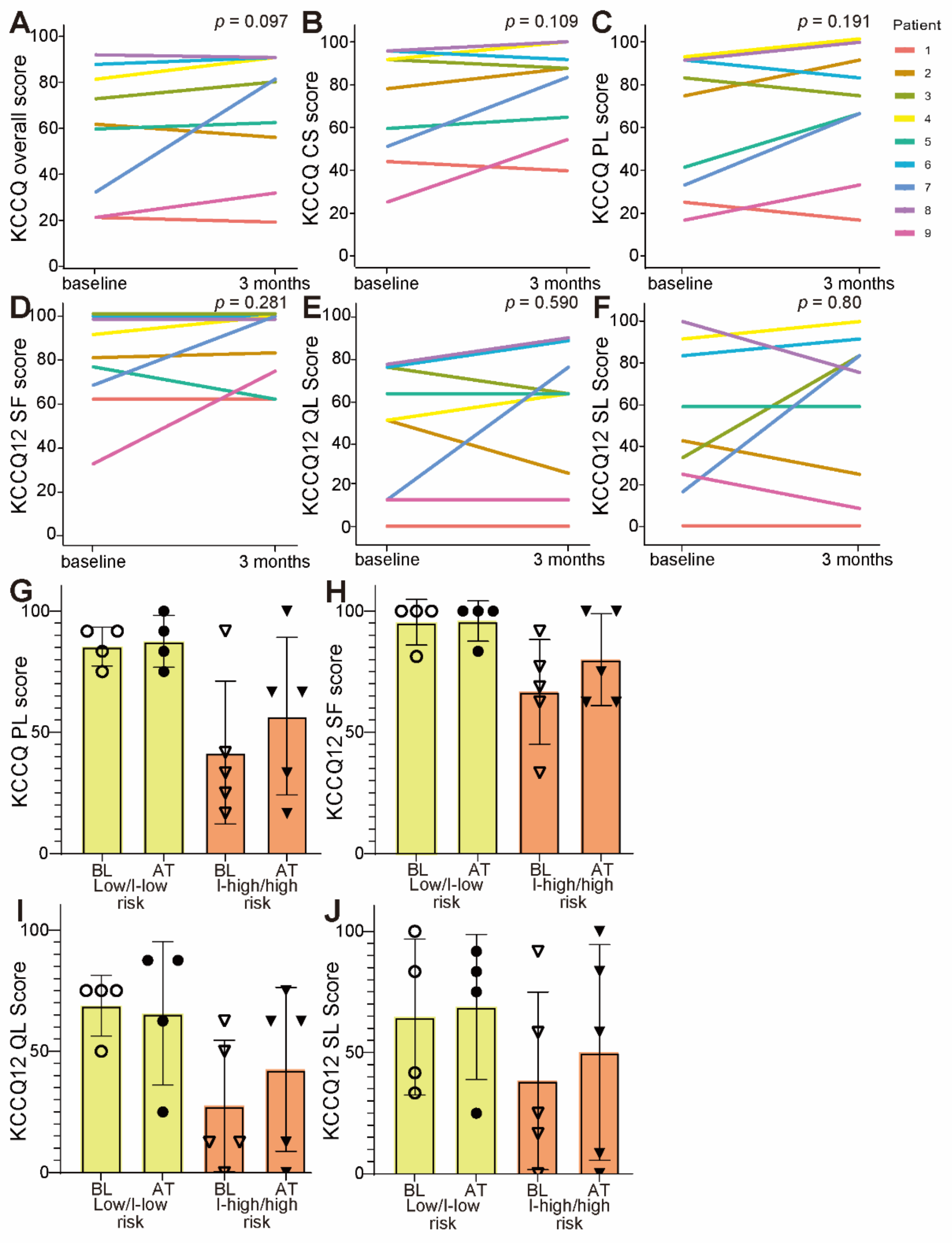


**Figure S2.** Changes in the Kansas City Cardiomyopathy Questionnaire-12 (KCCQ-12) score. Individual changes in the (A) KCCQ-12 overall summary score, (B) KCCQ-12 clinical summary (CS) score, (C) KCCQ physical limitation (PL) score, (D) KCCQ symptom frequency (SF) score, (E) KCCQ quality of life (QL) score, and (F) KCCQ symptom limitation (SL) score before and after inhaled treprostinil treatment. (G–J) Changes in KCCQ scores were compared between low/intermediate-low (I-low)-risk patients (yellow boxes) and intermediate-high (I-high)/high-risk patients (orange boxes). AT, after treat-ment; BL, baseline.
